# Longitudinal Surveillance and Risk Assessment of Resistance in *Escherichia coli* to Enrofloxacin from A Large-Scale Chicken Farm in Hebei, China

**DOI:** 10.3390/antibiotics10101222

**Published:** 2021-10-08

**Authors:** Kaixuan Guo, Yue Zhao, Luqing Cui, Zhengzheng Cao, Fan Zhang, Xiangru Wang, Zhong Peng, Jiawei Feng, Tianyu Hu, Menghong Dai

**Affiliations:** 1 The Cooperative Innovation Center for Sustainable Pig Production, College of Veterinary Medicine, Huazhong Agricultural University, Wuhan 430070, China; hzaugkx0612@webmail.hzau.edu.cn (K.G.); yue1226@webmail.hzau.edu.cn (Y.Z.); cuiluqing@webmail.hzau.edu.cn (L.C.); Mrczz180@webmail.hzau.edu.cn (Z.C.); zhangxiaofan@webmail.hzau.edu.cn (F.Z.); wangxr228@mail.hzau.edu.cn (X.W.); pengzhong@mail.hzau.edu.cn (Z.P.); fjw@webmail.hzau.edu.cn (J.F.); hty1996@webmail.hzau.edu.cn (T.H.); 2 MOA Key Laboratory of Food Safety Evaluation/National Reference Laboratory of Veterinary Drug Residue (HZAU), Huazhong Agricultural University, Wuhan 430070, China

**Keywords:** *Escherichia coli*, broiler farms, four breeding cycles, drug resistance, PMQRs, risk analysis

## Abstract

The purpose of this study was to investigate the changes of resistance phenotype and plasmid-mediated quinolone resistance genes (PMQRs) in *Escherichia coli* (*E. coli*) during enrofloxacin (ENR) administration in different breeding cycles. In 2020, 983 strains of *E. coli* were isolated from different samples in different cycles at the broiler farm with the largest single batch of slaughter capacity in Hebei Province, China. All samples were from chicken, environmental, and human sources. The sensitivity of the isolates to various antibiotics was determined by broth microdilution method. The findings of this study include: (1) the total isolation rate of *E. coli* in the four cycles was 63.83% (983/1540); (2) the average resistance rate of *E. coli* from 1-day-old chickens to enrofloxacin was as high as 75% in each cycle, and with the use of enrofloxacin, the resistance rate of *E. coli* from chickens gradually increased to 100%; (3) 107 strains of *E. coli* randomly selected from different cycles and sources demonstrated the multi-drug resistance phenotypes. The highest resistance rate was doxycycline (100%), and the lowest was erythromycin (54.21%); (4) the detection rate of PMQRs of *E. coli* from chickens in different cycles were always higher than that from environmental and human. In particular, the PMQRs pollution rate of chicken seedlings in each cycle were generally higher than that of other sources; (5) We used SPSS software to analyze the Kendall rank correlation of the experimental data. The resistance of *E. coli* isolated from this farm to ciprofloxacin (CIP) may increase along with the increase of resistance to enrofloxacin (Kendall’s tau-b = 0.190, *p* = 0.021). All these data highlight the serious problem of bacterial resistance in this farm. Therefore, it is urgent to provide guidance for the prevention and control of colibacillosis and drug resistance in this farm.

## 1. Introduction

As the pathogen of zoonotic diseases, animal-derived bacteria have always been one of the burdens affecting human health in the world. The development of antibiotic resistance and the lack of effective antibacterial drugs to prevent and treat bacterial diseases are the main challenges facing animal health [1]. Among them, *E. coli*, as a commensal bacterium in the intestinal tract of humans and warm-blooded animals, can enter the environment through feeding, excretion and other means [2]. According to statistics, *E. coli* has about 1020 populations and kills more than 2 million people every year through on-site or extra-intestinal diseases [3]. In developing countries, the prevalence of enterotoxigenic *E. coli*, enteroinvasive *E. coli*, enteropathogenic *E. coli* and enteroaggregative *E. coli* is an important reason that causes diarrhea in children and affects the development of animal husbandry. The most common enteropathogenic *E. coli* strain in developed countries is Shiga toxin-producing *E. coli*/enterohemorrhagic *E. coli* [4]. Large-scale population genetic studies have shown that the diversity and complexity of *E. coli* in different environments are affected by many environmental factors [5].

As an important indicator of contamination in animal and human life, the number of drug-resistant strains of *E. coli* has been increasing in recent years. Its drug resistance spectrum is complex and multi-drug resistance is serious [6,7]. This situation poses a serious threat to human and public health. Most outbreaks of human colibacillosis are related to food contamination. As chicken meat is the second meat consumable after pork in China, pollution mainly occurs in the links of breeding, slaughter, and product processing. China is a big country in breeding livestock and poultry in the world. Hebei Province, as one of the main producing areas of broilers in China, has a large scale of breeding, high density of breeding, and is also seriously affected by pathogenic *E. coli*. The study by Duan et al. reported that *E. coli* can survive widely in the chicken coop and its surrounding environment [8]. Therefore, this study selected a broiler breeding demonstration base in Hebei Province for *E. coli* resistance monitoring. There are at least four breeding cycles in the farm every year, and the medication mode of each cycle is basically the same. The farm had a long-term background of the use of fluoroquinolones. To prevent chickens from intestinal diseases, the farm mixed enrofloxacin with water to drink when the chickens were 9–12 days old. Therefore, this study focused on monitoring the resistance of chicken, environmental, and human *E. coli* to enrofloxacin during the four breeding cycles of the farm. The surveillance results can provide guidance for the prevention and control of *E. coli* disease and drug resistance in this farm, to reduce the mortality rate of chickens in this farm and improve the use rate of feed. This study will transmit correct and accurate breeding concepts for the farm, provide scientific and reasonable broiler raising methods, and contribute to the further improvement of the economic benefits of the farm.

## 2. Results

### 2.1. The Isolation Rate of E. coli in Four Breeding Cycles

Among the total of 1540 samples, the average separation rate of *E. coli* in the third breeding cycle (275/400, 68.75%) is highest (Table 1). The average isolation rate of *E. coli* from chicken in the four cycles was as high as 74.72% (538/720) (Table 1). The average isolation rate of *E. coli* in four breeding cycles of environmental source samples was the lowest (Table 1). The average separation rate of *E. coli* was highest in feed (65%) (Table 2). In the feed of the third cycle, the isolation rate of *E. coli* was as high as 90.00% (Table 2). In this study, the average separation rates of *E. coli* in feces and fecal water during the four cycles were 48.34% and 63.33%, respectively (Table 2). Our investigation shows that the average separation rate of *E. coli* in each cycle of biological media samples was 53.33% (Table 2). In addition, human beings are important spreaders of bacteria. In this study, the average isolation rate of *E. coli* from broiler breeders in the four cycles was 77.50% (Table 1). In the fourth cycle, the isolation rate of *E. coli* from human was even as high as 100% (Table 2). This is closely related to the personal hygiene of the breeders.

### 2.2. The Sensitivity of E. coli to Enrofloxacin and Other Antibiotics

**Figure 1:**
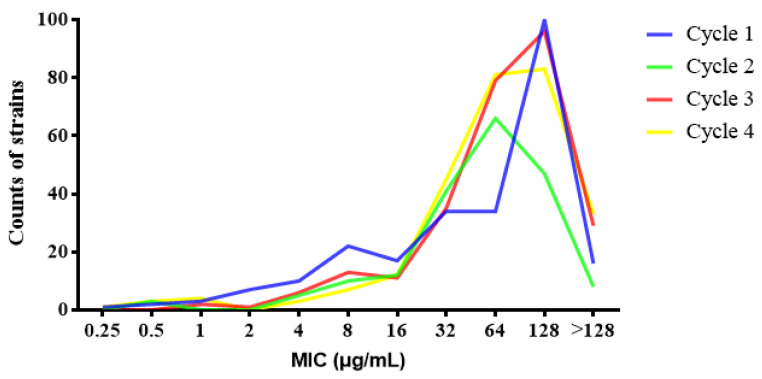
MIC value distribution of 983 strains of *E. coli* to enrofloxacin in four breeding.

The resistance rate of 983 test strains to ENR was as high as 97.25% (956/983). The MIC value of most *E. coli* is between 32–128 μg/mL, accounting for 75.38% (741/983). Only 10 strains of *E. coli* are sensitive to ENR (MIC ≤ 1 μg/mL) (Figure 1). The average resistance rates of *E. coli* isolated in each breeding cycle to enrofloxacin were 95.20%, 98.44%, 98.90% and 95.20%, respectively (Table 3). The resistance rate of *E. coli* isolated from 1-day-old chicken seedlings in each breeding cycle to ENR was more than 75% (Table 3). In addition, with the use of ENR in chickens between 9–12 days of age, the resistance rate of *E. coli* from chickens to ENR would gradually increase to 100.00% (Table 3). The average resistance rate of *E. coli* from environmental sources in each breeding cycle to ENR was as high as 95% (Table 3). There was no significant difference in the resistance rate of *E. coli* to enrofloxacin between different breeding cycles, and all showed high levels of resistance. However, it should be noted that because the total number of *E. coli* strains tested in each link is small, small changes will cause differences in the statistics of drug resistance rates. In cycle 2, only one strain of *E. coli* is sensitive to enrofloxacin, which leads to a decrease in resistance level one day after medication in comparison to other cycles. However, only one strain of *E. coli* is not representative and does not explain the problem. In general, these *E. coli* strains maintained a high resistance rate to enrofloxacin.

**Figure 2:**
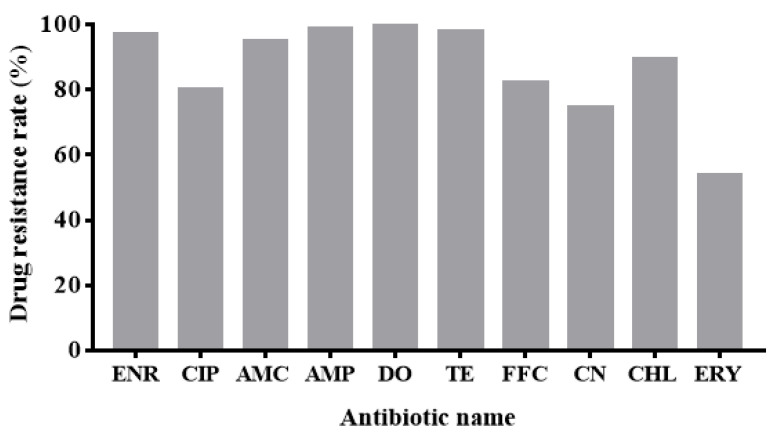
The resistance rates of 107 strains of *E. coli* to 10 kinds of antibiotics.

**Figure 3:**
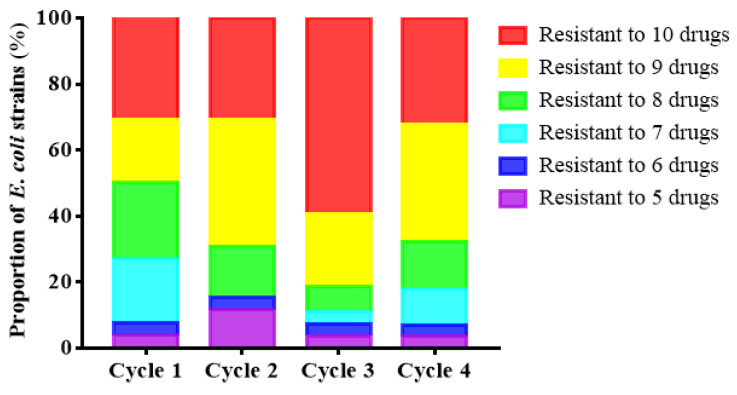
Resistance rates of 107 strains of *E. coli* to different antibiotics in different.

**Figure 4:**
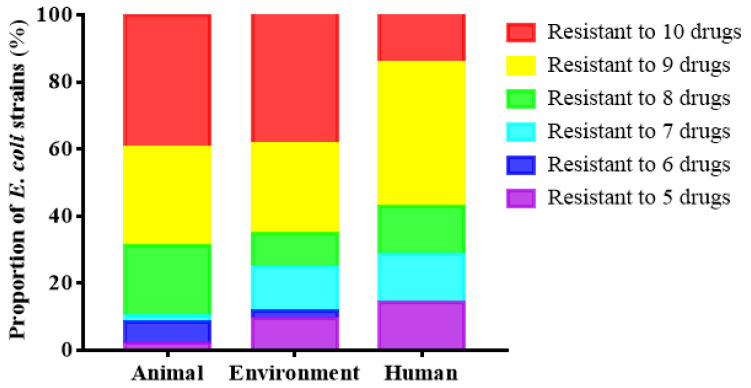
Resistance rates of 107 strains of *E. coli* from different sources to different antibiotics.

The non-repetitive 107 strains randomly selected from 983 *E. coli* strains had a serious problem of multi-drug resistance to the 10 antibiotics tested. Specifically, 107 strains of *E. coli* had a positive effect on enrofloxacin, ciprofloxacin, amoxicillin/clavulanic acid (AMC), ampicillin (AMP), doxycycline (DO), tetracycline (TE), florfenicol (FFC), gentamicin (CN), chloramphenicol (CHL) and erythromycin (ERY) resistance rates were 98.13% (105/107), 80.37% (86/107), 92.70% (99/107), 99.07% (106/107), 100.00% (107/107), 98.13% (105/107), 82.24% (88/107), 74.77% (80/107), 89.72% (96/107) and 54.21% (58/107), respectively (Figure 2). In terms of cycle, the resistance of strains in the first cycle is relatively mild. The resistance of the third cycle strains is more serious (Figure 3). According to the source of strains, the multi-drug resistance of *E. coli* from chicken is the most serious. Its resistance to 8 or more antibiotics accounted for as high as 87.49% (Figure 4).

### 2.3. Correlation Analysis among Multiple Antibiotic Resistance

This study found that the *E. coli* isolated from the broiler farm had a significant positive correlation to ENR and CIP resistance (Table 4). Even the farm has never used CIP as a medicine for clinical prevention and treatment of chicken diseases in each breeding cycle. However, the resistance rate of 107 *E. coli* strains isolated from the site to CIP was as high as 80.37%. The resistance of *E. coli* to CIP may increase with the increase of its resistance to ENR. Similar results include DO and TE, both of which belong to the tetracycline class, and FFC and CHL, which belong to the same class of chloramphenicol (Table 4). The difference was that ERY, which belongs to macrolides, and CN, which belongs to aminoglycosides, showed a strong positive correlation with drug resistance (Table 4). It should be noted that this result can only represent the correlation between the ten antibiotics tested, and there may also be a stronger correlation between an untested antibiotic and a certain drug tested in this study.

### 2.4. Detection of Plasmid-Mediated Fluoroquinolone Resistance Genes in E. coli

**Figure 5:**
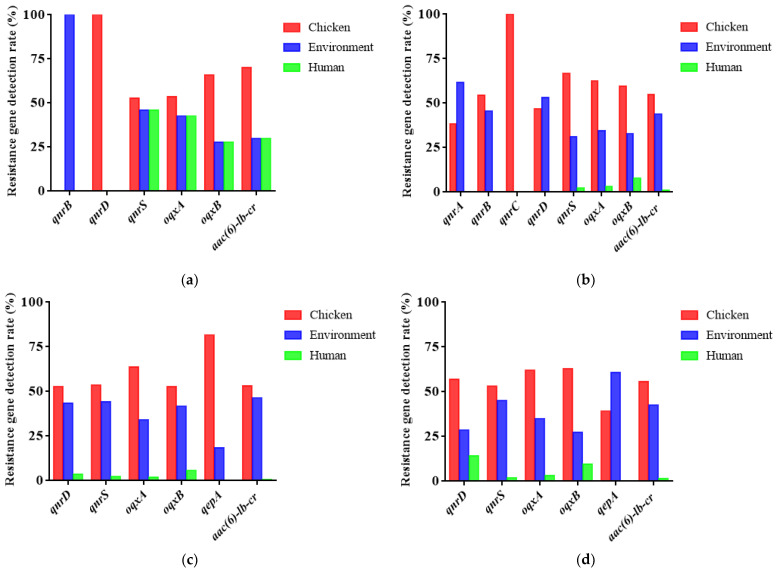
Detection rate of each drug resistance gene in *E. coli* from different cycles and sources. (**a**–**d**) respectively represent the first cycle to the fourth cycle.

**Figure 6:**
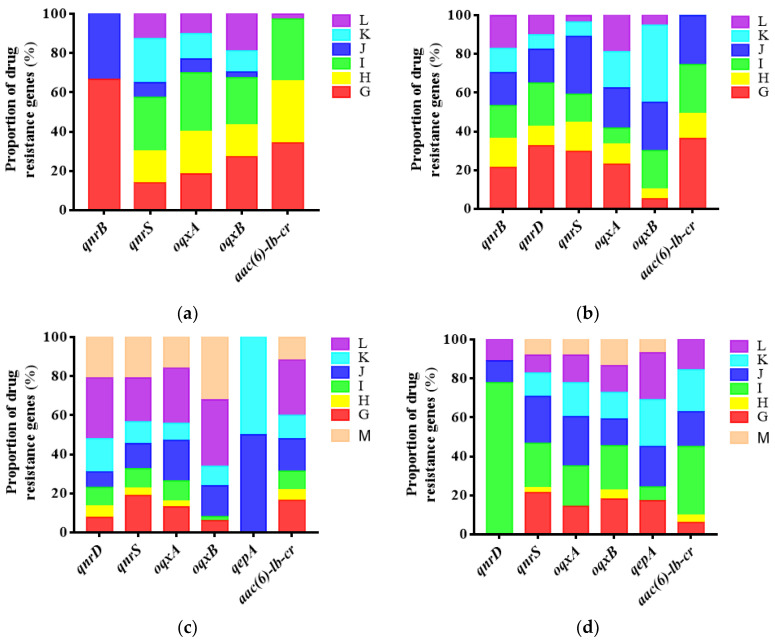
The proportion of the detection rate of antibiotic-resistant genes in *E. coli* from environmental sources in different cycles. (**a**–**d**) respectively represent the first cycle to the fourth cycle. M: Biological media; G: Feed; H: Water source; I: Fecal water; J: Dust; K: Feces; l: Tools.

**Figure 7:**
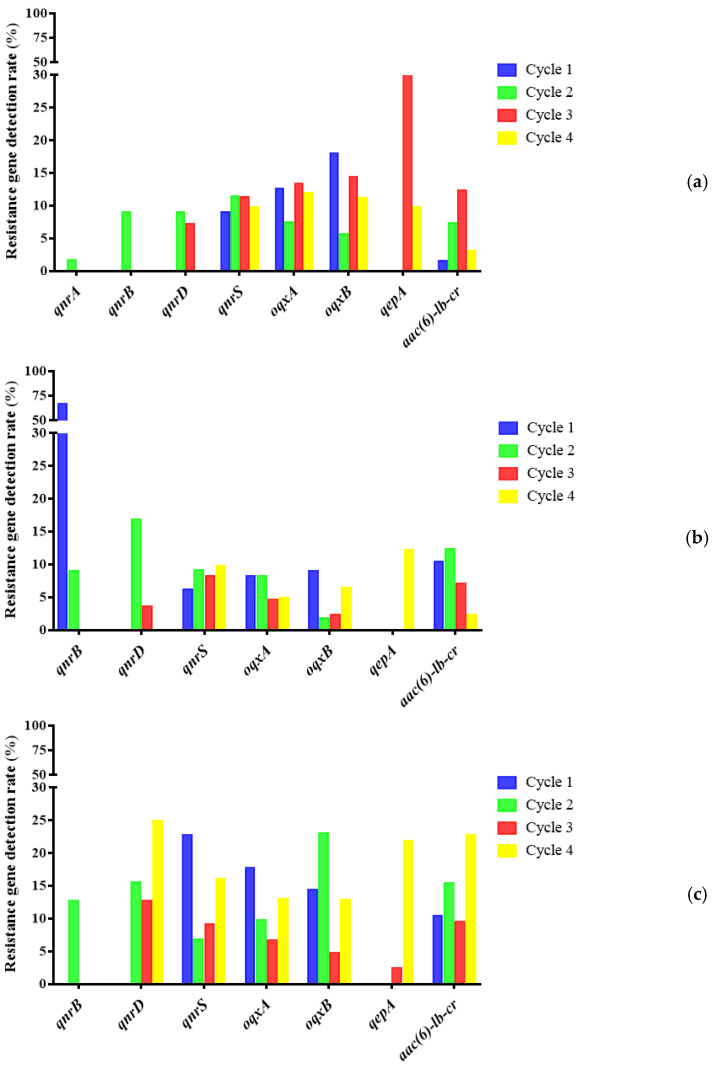
(**a**) The proportion of strains with positive drug resistance genes from chicken seedlings in different cycles. (**b**) Proportion of all positive strains of each drug resistance gene from feed in different cycles. (**c**) Proportion of all positive strains of each drug resistance gene from fecal water and feces in different cycles.

Six plasmid-mediated fluoroquinolone resistance genes were detected among 246 strains of *E. coli* in the first cycle. The detection rate of *qnrS* was the highest, which was 71.54% (176/246). Eight PMQRs were detected among 192 strains of *E. coli* in the second cycle. The detection rate of *aac(6)-lb-cr* was the highest, which was 84.38% (162/192). Six PMQRs were detected among 275 strains of *E. coli* in the third cycle. The detection rate of *qnrS* was the highest, which was 82.91% (228/275). Six PMQRs were detected among 272 strains of *E. coli* in the fourth cycle. The detection rate of *oqxA* was the highest, which was 68.15% (84/270) (Table 5) (*p* < 0.05). It is noteworthy that in different cycles, the detection rate of resistance genes of chicken *E. coli* was always dominant compared with environmental and human sources (Figure 5). The detection rate of *E. coli* resistance genes in feed, fecal water, and feces accounted for a higher level in the overall environmental samples. (Figure 6). Specific analysis of strains from chicken seedlings, feed, feces, and fecal water show that *qnrS*, *oqxA*, *oqxB*, and *aac(6)-lb-cr* could be detected in the strains from different sources in different cycles. They are relatively dominant genes (Figure 7). These resistance genes have a high detection rate in chicken-derived strains, especially chicken seedlings strains. In addition, *qnrA* only appeared in chicken-derived strains in the second breeding cycle. In addition, *qnrA* can also be detected in some chick seedlings. Maybe *qnrA* was brought into the farm by chicken seedlings (Figure 7). *QepA* gene appears in strains of the third and fourth cycles. The detection rate of chicken seedling strains in the third cycle was 34.88%. The detection rate of feces and fecal water strains did not exceed 5%, and it was not detected in other links. However, the detection rate of this gene in the fourth cycle chicken seedlings was 9.76%. The detection rate in feed, feces and fecal water all exceeded 15%. It is possible that *qepA* was brought into the farm by the chicken seedlings of the third cycle, and horizontal transmission occurred in the farm (Figure 7). *QnrB* is not present in the chick strains of the first cycle. It is only detected in feed and dust strains, but the detection rate is extremely low. However, *qnrB* can be detected in every link of the second cycle. It is possible that the feed of the first cycle was the source of *qnrB* transmission, and then horizontal transmission occurred in the field (Figure 7). *QnrD* does not exist in the first cycle chicken seedlings and environmental strains. It is only detected in chicken-derived strains in the process of medication, and can be detected in all phases of the subsequent cycles. It is possible that *qnrD* was induced during the first cycle of medication and then spread (Figure 7).

## 3. Discussion

### 3.1. The Prevalence of E. coli in Broiler Farms

In all breeding cycles, the isolation rate of *E. coli* in the third cycle is higher. Because the sampling time of this cycle is in July, which are hot and rainy. In the summer in northern China, the overall temperature of the chicken house is relatively high and difficult to control, and the environment is relatively humid. Studies have shown that in the season of high temperature, the carrying rate of pathogenic bacteria in animals is higher [9]. Comparing different sources, chicken-derived *E. coli* has the highest isolation rate. It was possibly because the chicken seedlings have already carried pathogenic bacteria during the hatching stage of the breeder farm. Many studies have also found this phenomenon. For example, Zhao et al. investigated the chicken seedlings in the hatchery and found that some chicken seedlings carried typical avian pathogenic *E. coli*. They also found that the same strains in the same hatchery may come from the vertical transmission of parent groups [10]. In addition, studies had shown that *E.coli* strains isolated from the intestine of healthy chickens and from their environment are phylogenetically similar to pathogenic *E. coli* (APEC) strains that have been isolated from coliseptisemic chickens [11]. Therefore, we must strictly control the quality of chicken seedlings to reduce the introduction of bacterial pathogens. When selecting chicken seedlings, they should be purchased from the regular farms with breeding livestock, poultry certificate, and animal epidemic prevention condition certificate. Farms should choose healthy, energetic, lively, fluffy, and bright chicken seedlings. The healthy chickens should be vaccinated in time. After the chicken seedlings produces the corresponding antibodies, the occurrence and prevalence of infectious diseases in chickens will be reduced.

The high separation rate of feed source *E. coli* in environmental samples further illustrates the importance of feed in the broiler feeding process. The study by Davis et al. showed that the pulsed field gel electrophoresis (PFGE) spectrum of *E. coli* O157:H7 isolated from a farm feed sample is very similar to the spectrum of the later isolated from the farm’s feces. This provides evidence for the role of feed in the spread of *E. coli* O157:H7 [12]. In addition, feces, water, soil, flies, and mice are all common vectors of *E. coli*. Since the farm has done a good job in the purification of chicken drinking water and the disinfection of the ground and air in the chicken house, the isolation rate of *E. coli* in water and dust samples is relatively low. Experiments by Chuppava et al. proved that the feces of broiler chickens carry drug-resistant *E. coli* with multiple drug-resistant genes and spread in the environment around the chicken house [13]. Colomer-Lluch and Selvam’s research also confirmed this view [14,15]. When untreated animal manure is used as fertilizer in farmland or discharged into rivers with sewage, it will cause irreversible harm to the natural environment [16]. High separation rate of *E. coli* in feces and fecal water samples reminds that the farm should clear the manure on the conveyor belt in the house in time, and treat the manure water accumulated after cleaning in a timely manner. Flies are often an easily neglected pathogen transmission vector. In chicken houses, a large number of flies often attach to chicken body, excrement, breeding tools, and feed surface, which is often conducive to the colonization and transmission of *E. coli* [17]. High separation rate of *E. coli* in biological vector samples also reminds the farm that it should strengthen the control and killing of flies and mosquitoes in the house to eliminate potential threats that may be caused by biological vectors.

In addition, human beings are important spreaders of bacteria. The results of this experiment suggest that the maintenance of personal hygiene of our farmers is very important. Adenipekun et al. confirmed that drug-resistant *E. coli* can spread to humans through the food chain, and most of these people are engaged in poultry breeding or sales [18]. Therefore, the farm should strengthen the hygiene and health management of the breeders and maintain good hygiene habits to block the spread of pathogenic bacteria.

### 3.2. Epidemic Characteristics of E. coli Resistance and Multi-Drug Resistance in Broiler Farms

This study found that the resistance of *E. coli* is serious in the farm, especially the resistance of *E. coli* isolated from chicken seedlings. It is speculated that the high resistance rate of *E. coli* from chicken-derived to ENR at the stage of chicken seedlings entering the farm may be caused by the vertical transmission of the parents [10]. Nishikawa et al. proved that *E. coli* carrying the PMQR gene can cause resistance to similar drugs under the selective pressure of fluoroquinolones [19]. Therefore, the high resistance rate of *E. coli* isolated in the later period to ENR may be related to the frequent use of ENR for prevention and control by the farm for a long time and the changes in the chicken house environment over time. In this case, the farm should actively change the way of medication in each breeding cycle. Farms should take reasonable preventive measures, or use interval and shuttle medication methods under necessary conditions to reduce the frequency of drug resistance.

The result of the high multi-drug resistance may be caused by the unreasonable use of various antibiotics in the farm in each breeding cycle. According to the investigation, the farm used a large amount of AMC, FFC, DO, and ERY in each breeding cycle. This also caused the isolated *E. coli* to have a high resistance rate to these and their similar drugs. The investigation by Han T et al. also proved that drug-resistant bacteria in animals and the environment continued to increase with the use of drugs during the feeding cycle [20]. This had also led to the widespread distribution of resistance genes and the increase in overall bacterial resistance. The farm should look for alternative therapies for clinical treatment of chicken diseases. We consider that vaccines and Chinese medicine can be used to prevent and treat various diseases. For example, Koutsianos D et al. found that birds that have been vaccinated with commercial live and inactivated vaccines can be better protected from pathogenic bacteria [21]. Moreover, the multi-drug resistance of environmental and human *E. coli* is also quite serious. Under the selective pressure of heavy metals and fungicides in the environment, the spread of arthropods, the long-term contact with animals or environment carrying drug-resistant strains and the normal physiological function of the human body is seriously disrupted, bacteria will produce different degrees of drug resistance [22,23]. Van Boeckel et al. reported that drug-resistant bacteria from animals can not only spread widely in the environment through feces, but also easily spread to humans through the food chain [24]. More investigations have also proved that multi-drug-resistant *E. coli* isolated from environmental sources and human sources are mostly caused by the horizontal transmission of resistant strains between the animals, environment, food and people [25]. Therefore, more attention should be paid to improve the environmental sanitation of the farm and the personal health of the breeder. The breeding farm should conduct regular physical examinations on the breeders. The farm must ensure that the living area and the chicken coop are clean and tidy to prevent the generation and spread of pathogens. In addition, the cultivation of biosafety awareness of farmers is also particularly important.

### 3.3. Risk Assessment of Drug Resistance in Broiler Farms Based on SPSS Nonparametric Correlation Analysis

Fluoroquinolones, tetracyclines, and other antibiotics have been frequently used in large-scale farms in China for a long time, which not only reduce the occurrence and spread of population diseases, but also lead to the thorny problem of drug resistance or multi-drug resistance. Significant positive correlation results between ENR and CIP possibly because they belong to the fluoroquinolone antibiotics, and CIP is the main active metabolite when ENR works. After *E. coli* was resistant to ENR, it developed cross-resistance to CIPs with similar structures and properties [26,27]. Moreover, the metabolism of ENR and CIP in animals is relatively slow, and drug residues are easy to occur, resulting in the increase of drug resistance of pathogens in organisms. This fully explains the phenomenon that the resistance rate of *E. coli* isolated from this farm to ENR gradually increases with the age of broilers. Similar results include DO and TE, FFC and CHL both showed strong positive correlation in correlation analysis [28,29]. We speculate that there are similar mechanisms of action and drug resistance among several drugs belonging to a certain class of antibiotics. Thus, the resistance of pathogenic microorganisms to these antibacterial drugs is positively correlated. However, this phenomenon needs further research to prove it. The ERY and CN, which belongs to different antibiotics, showed a strong positive correlation with drug resistance. This phenomenon may be because the strains carrying the CN resistance gene can combine and transfer with the strains carrying the ERY resistance gene. So that pathogens carry two kinds of resistance genes at the same time [30]. Studies have also shown that a plasmid with high homology to blaCTX-M carries a variety of antibiotic resistance genes at the same time [31]. It can be seen that different kinds of antibiotics have a positive correlation with drug resistance, which may be caused by the horizontal transfer of drug resistance genes between the tested strains or the tested strains carrying resistance genes to multiple drugs at the same time. It should be noted that this result can only represent the correlation between the ten antibiotics tested, and there may also be a stronger correlation between an untested antibiotic and a certain drug tested in this study. Therefore, if the previous drug strategy is maintained, the bacteria isolated from the farm will only become more resistant to various antibiotics. This will not only threaten the growth of chickens, but also cause irreversible harm to the environment and people. In the end, it may cause serious consequences that no medicine is available.

### 3.4. Epidemic Characteristics of Plasmid-Mediated Fluoroquinolone Resistance Genes in Broiler Farms

The results showed that the pollution rate of PMQRs in every cycle of chicken seedlings in this farm is extremely high. Moreover, the long-term medication pressure of ENR may lead to an increase in the detection rate of some PMQRs. At the same time, PMQRs in the environment are most likely caused by the horizontal spread of PMQRs-carrying strains in chicken sources. Many studies have proved that chicken seedlings may carry PMQRs during the hatching stage of the breeder farm, and spread horizontally or vertically after entering the farm [32,33]. When in an environment with drug selection pressure, it is conducive to select PMQRs and spread. In addition, after genes such as *oqxA* and *oqxB* are specifically expressed in *E. coli*, the MIC of quinolones against *E. coli* can be increased by more than 8 times. This explains well the reason the resistance rate of *E. coli* increased during the administration of enrofloxacin in this study, and eventually reached 100.00%. In broiler production, other ways, such as transmission by flies or horizontal gene transfer of other bacteria, may be more important than vertical transmission [34]. However, no matter what kind of transmission method, it may promote the bacterial resistance rate or the detection rate of drug resistance genes to gradually increase with the increase of the age of broilers.

## 4. Materials and Methods

### 4.1. Collection of Chicken, Environmental, and Human Samples

In 2020, we collected a total of 1540 samples of chicken, environmental, and human sources from a large-scale broiler farm in Handan City, Hebei Province during four breeding cycles. The farm has at least four breeding cycles each year, and the respective cycles are roughly in January to March, April to June, July to September, and October to December. Each breeding cycle is about 42 days, and the medication mode of each cycle is basically the same. The farm has a long-term background of using fluoroquinolones. Generally, broilers drink ENR mixed with water at the age of 9–12 days. The purpose is to prevent intestinal diseases, such as colibacillosis. In addition, amoxicillin, florfenicol, erythromycin, doxycycline and other kinds of antibiotics were also used in broilers at different ages. Among the 1540 samples, 720 chicken samples came from cloaca of healthy chickens. A total of 780 environmental source samples were collected from feed, drinking water, feces, dust, fecal water, breeding tools, and biological media in the chicken house. A total of 40 human samples came from the feces of breeding workers. All the samples were collected with disposable sterile swabs and transported to the laboratory through cold chain transportation in time for the next experiment.

### 4.2. Isolation and Identification of E. coli

After the collected samples were enriched, the bacterial solution was inoculated on LB agar plates for overnight culture. The white and round colonies were inoculated on LB agar for proliferation, and then purified on MacConkey agar medium. Subsequently, the pink, round raised, smooth, moist, and neatly edged colonies on MacConkey’s medium were inoculated on the eosin-methylene blue agar medium for re-identification. Finally, the colonies of suspected *E. coli* were picked (the colony is black and slightly convex, the edges are neat, the surface is smooth, moist, and has a metallic luster) for PCR identification. We used bacterial lysate as a template for PCR amplification. The specific primers were *16S rDNA* (5′-GAAGCTTGCTTCTTTGCT-3′and 5′-GAGCCCGGGGATTTCACAT-3′) [35] to confirm that the tested bacteria is *E. coli*. The technological process was under the following amplification conditions: 95 °C for 3 min; followed by 34 cycles of 95 °C for 15 s, 55 °C for 15 s, and 72 °C for 1 min; and extension at 72 °C for 5 min. The amplified products were sent to Wuhan Qingke Biological Technology Limited Company for sequencing to confirm the PCR results. The sequencing results of the strains were compared on the National Center for Biotechnology Information website to ensure that the homology between these strains and *E. coli* was above 97%.

### 4.3. Antimicrobial Susceptibility Testing

The microbroth dilution method was used to determine the MIC of 983 strains of *E. coli* to ENR, and their drug resistance was described according to the Clinical Laboratory Standards Institute Guidelines (CLSI) [36]. The MICs of 107 strains of *E. coli* randomly selected from different cycles and sources to 10 kinds of antibiotics were determined by the same method, including enrofloxacin, ciprofloxacin, amoxicillin/clavulanic acid, ampicillin, florfenicol, doxycycline, gentamicin, tetracycline, chloramphenicol, and erythromycin. *E. coli* ATCC 25922 was served as a quality control strain for susceptibility tests.

### 4.4. Correlation Data Analysis of Multiple Antibiotic Resistance Levels

SPSS 17.0 software was used to analyze the Kendall rank correlation on all MIC results of 107 strains of *E. coli*. Kendall correlation belongs to nonparametric test, which is a quantity that reflects the degree of correlation between ordinal variables [37]. When using this correlation analysis method, the total distribution of variables is not required to be normal, and the sample size is not required to be greater than 30 [38]. * *p* ≤ 0.05 means significant correlation, * *p* ≤ 0.01 means highly significant correlation. According to the results, we analyzed the drug resistance correlation of *E. coli* under the fixed medication mode, and judged whether the long-term use of enrofloxacin affected the drug resistance level of *E. coli* to different drugs.

### 4.5. Plasmid-Mediated Fluoroquinolone Resistance Gene Detection

After isolates were confirmed to be *E. coli*, all of them were tested using the primer pairs (5′ to 3′) for *qnrA* (TCAGCAAGAGGATTTCTCA/GGCAGCACTATTACTCCCA), *qnrB* (GATCGTGAAAGCCAGAAAGG/ACGATGCCTGGTAGTTGTCC), *qnrC* (GGGTTGTACATTTATTGAATC/TCCACTTTACGAGGTTCT), *qnrD* (CGAGATCAATTTACGGGGAATA/AACAAGCTGAAGCGCCTG), *qnrS* (ACGACATTCGTCAACTGCAA/TAAATTGGCACCCTGTAGGC), *oqxA* (CTCGGCGCGATGATGCT/CCACTCTTCACGGGAGACGA), *oqxB* (TTCTCCCCCGGCGGGAAGTAC/CTCGGCCATTTTGGCGCGTA), *qepA* (GCAGGTCCAGCAGCGGGTAG/CTTCCTGCCCGAGTATCGTG) and *aac(6)-lb-cr* (TTGCGATGCTCTATGAGTGGCTA/CTCGAATGCCTGGCGTGTTT) by PCR [39,40]. The above genes were measured to monitor and analyze the prevalence of plasmid-mediated fluoroquinolone resistance genes in the farm.

## 5. Conclusions

In short, this research warns us that although changing the way of using antibiotics or improving the breeding environment will have a certain inhibitory effect on the development of drug resistance, the top priority thing is to control the quality and source of chicken seedlings. Only by controlling the source of drug resistance can the benign development of the broiler-related industry chain and the health and safety of human food public health be more effectively guaranteed. All in all, solving the problem of antibiotic resistance is not a matter of overnight. It requires the joint efforts of all personnel.

## Figures and Tables

**Table 1:** Isolation rate of *E. coli* in four breeding cycles (unit: %).

Sampling Time	Chicken	Environment	Human	Total
Cycle 1 1.12–1.26	72.78(131/180)	65.00(117/180)	80.00(8/10)	66.49(246/370)
Cycle 2 4.02–4.16	58.33(105/180)	45.56(82/180)	50.00(5/10)	51.89(192/370)
Cycle 3 7.14–7.28	81.67(147/180)	57.14(120/210)	80.00(8/10)	68.75(275/400)
Cycle 4 9.12–9.26	86.11(155/180)	50.00(105/210)	100.00(10/10)	67.50(270/400)
Total	74.72(538/720)	54.36(424/780)	77.50(31/40)	63.83(983/1540)

Note: The brackets indicate the ratio of the number of resistant bacteria to the total number of strains.

**Table 2:** Isolation rate of *E. coli* from different sources in four cycles (unit: %).

Cycle	Chicken						Environment							Human
	A	B	C	D	E	F	G	H	I	J	K	L	M	N
1	80.00	86.67	66.67	70.00	53.33	80.00	50.00	63.33	83.33	33.33	80.00	80.00		80.00
2	40.00	66.67	73.33	76.67	43.33	50.00	66.67	23.33	56.67	46.67	36.67	43.33		50.00
3	96.67	86.67	80.00	90.00	50.00	86.67	90.00	16.67	33.33	63.33	36.67	86.67	73.33	80.00
4	93.33	60.00	96.67	93.33	86.67	86.67	53.33	6.67	80.00	93.33	40.00	43.33	33.33	100.00
Four-cycle average	77.50	75.00	79.17	82.50	58.33	75.84	65.00	27.50	63.33	59.17	48.34	63.33	53.33	77.50

Note: A: Chicken seedlings (1 day old); B: The day before medication (8 days old); C: First day of medication (9 days old); D: Third day of medication (11 days old); E: One day after medication (13 days old); F: Three days after medication (15 days old); G: feed; H: water source; I: Fecal water; J: Dust; K: Feces; L: Tool; M: Biological vector; N: Breeders. The samples of environment and human and chicken seedlings were collected at the same time. Blank data means that no samples had been collected in this link.

**Table 3:** Resistance rate of *E. coli* isolated in four breeding cycles to enrofloxacin (unit: %).

Cycle	Chicken Seedlings	The Day before Medication	First Day of Medication	Third Day of Medication	One Day after Medication	Three Days after Medication	Environment	Human	Overall Average
1	79.17	88.46	100.00	100.00	100.00	100.00	96.21	100.00	95.20
2	100.00	100.00	100.00	100.00	92.31	100.00	97.56	100.00	98.44
3	93.10	96.15	100.00	100.00	100.00	100.00	100.00	100.00	98.90
4	89.29	95.00	96.55	100.00	100.00	100.00	97.14	100.00	95.20

**Table 4:** Nonparametric correlation analysis of antibiotic resistance.

			ENR	CIP	AMC	AMP	DO	TE	FFC	CN	CHL	ERY
**Kendall tau_b**	**ENR**	**Correlation coefficient**	1.000	0.190 ^*^	0.172	−0.065	0.034	−0.010	0.027	0.100	−0.054	0.015
		**Sig.(Double tail)**	.	0.021	0.071	0.498	0.693	0.913	0.762	0.235	0.550	0.862
		**N**	97	97	91	90	97	91	97	97	96	92
	**CIP**	**Correlation coefficient**	0.190 ^*^	1.000	0.079	0.008	0.034	0.104	0.284 ^**^	0.318 ^**^	0.174 ^*^	0.156
		**Sig.(Double tail)**	0.021	.	0.366	0.930	0.663	0.224	0.001	0.000	0.036	0.056
		**N**	97	107	101	100	107	101	106	106	105	101
	**AMC**	**Correlation coefficient**	0.172	0.079	1.000	−0.058	0.131	0.155	−0.126	0.028	−0.133	0.034
		**Sig.(Double tail)**	0.071	0.366	.	0.567	0.152	0.117	0.189	0.755	0.166	0.721
		**N**	91	101	101	95	101	97	100	100	99	96
	**AMP**	**Correlation coefficient**	−0.065	0.008	−0.058	1.000	0.138	0.064	0.006	0.032	0.079	0.026
		**Sig.(Double tail)**	0.498	0.930	0.567	.	0.136	0.523	0.955	0.724	0.416	0.781
		**N**	90	100	95	100	100	95	99	99	98	96
	**DO**	**Correlation coefficient**	0.034	0.034	0.131	0.138	1.000	0.349 ^**^	0.159	0.044	0.285 ^**^	0.231 ^**^
		**Sig.(Double tail)**	0.693	0.663	0.152	0.136	.	0.000	0.066	0.594	0.001	0.007
		**N**	97	107	101	100	107	101	106	106	105	101
	**TE**	**Correlation coefficient**	−0.010	0.104	0.155	0.064	0.349 ^**^	1.000	0.270 ^**^	0.073	0.187 ^*^	0.197 ^*^
		**Sig.(Double tail)**	0.913	0.224	0.117	0.523	0.000	.	0.004	0.410	0.047	0.032
		**N**	91	101	97	95	101	101	100	100	99	97
	**FFC**	**Correlation coefficient**	0.027	0.284 ^**^	−0.126	0.006	0.159	0.270 ^**^	1.000	0.224 ^**^	0.352 ^**^	0.312 ^**^
		**Sig.(Double tail)**	0.762	0.001	0.189	0.955	0.066	0.004	.	0.009	0.000	0.001
		**N**	97	106	100	99	106	100	106	106	104	100
	**CN**	**Correlation coefficient**	0.100	0.318 ^**^	0.028	0.032	0.044	0.073	0.224 ^**^	1.000	0.255 ^**^	0.385 ^**^
		**Sig.(Double tail)**	0.235	0.000	0.755	0.724	0.594	0.410	0.009	.	0.003	0.000
		**N**	97	106	100	99	106	100	106	106	104	100
	**CHL**	**Correlation coefficient**	−0.054	0.174 ^*^	−0.133	0.079	0.285 ^**^	0.187 ^*^	0.352 ^**^	0.255 ^**^	1.000	0.285 ^**^
		**Sig.(Double tail)**	0.550	0.036	0.166	0.416	0.001	0.047	0.000	0.003	.	0.001
		**N**	96	105	99	98	105	99	104	104	105	100
	**ERY**	**Correlation coefficient**	0.015	0.156	0.034	0.026	0.231 ^**^	0.197 ^*^	0.312 ^**^	0.385 ^**^	0.285 ^**^	1.000
		**Sig.(Double tail)**	0.862	0.056	0.721	0.781	0.007	0.032	0.001	0.000	0.001	.
		**N**	92	101	96	96	101	97	100	100	100	101

Note: ^*^ At 0.05 level (two tailed), the correlation was significant. ^**^ At 0.01 level (two tailed), the correlation was significant.

**Table 5:** Detection rate of drug resistance genes in different cycles of *E. coli* (unit: %).

Gene Name	Cycle 1	Cycle 2	Cycle 3	Cycle 4
*qnrA*	0	31.25(60/192)	0	0
*qnrB*	1.21(3/246)	57.29(110/192)	0	0
*qnrC*	0	0.05(1/192)	0	0
*qnrD*	1.63(4/246)	40.10(77/192)	40.00(110/275)	10.37(28/270)
*qnrS*	71.54(176/246)	45.31(87/192)	82.91(228/275)	60.00(162/270)
*oqxA*	64.23(158/246)	69.27(133/192)	70.55(194/275)	68.15(184/270)
*oqxB*	45.12(111/246)	27.08(52/192)	45.45(125/275)	22.96(62/270)
*qepA*	0	0	15.64(43/275)	15.19(41/270)
*aac(6)-lb-cr*	50.41(124/246)	84.38(162/192)	76.36(210/275)	47.04(127/270)

Note: The brackets indicate the ratio of the strains carrying the drug resistance gene to the total number of strains.

## Data Availability

Data are contained within the article.
